# Building a Pharmacogenomics Knowledge Model Toward Precision Medicine: Case Study in Melanoma

**DOI:** 10.2196/20291

**Published:** 2020-10-21

**Authors:** Hongyu Kang, Jiao Li, Meng Wu, Liu Shen, Li Hou

**Affiliations:** 1 Institute of Medical Information &Library Chinese Academy of Medical Sciences/Peking Union Medical College Beijing China; 2 Department of Biomedical Engineering School of Life Science Beijing Institute of Technology Beijing China

**Keywords:** pharmacogenomics, knowledge model, BERT–CRF model, named entity recognition, melanoma

## Abstract

**Background:**

Many drugs do not work the same way for everyone owing to distinctions in their genes. Pharmacogenomics (PGx) aims to understand how genetic variants influence drug efficacy and toxicity. It is often considered one of the most actionable areas of the personalized medicine paradigm. However, little prior work has included in-depth explorations and descriptions of drug usage, dosage adjustment, and so on.

**Objective:**

We present a pharmacogenomics knowledge model to discover the hidden relationships between PGx entities such as drugs, genes, and diseases, especially details in precise medication.

**Methods:**

PGx open data such as DrugBank and RxNorm were integrated in this study, as well as drug labels published by the US Food and Drug Administration. We annotated 190 drug labels manually for entities and relationships. Based on the annotation results, we trained 3 different natural language processing models to complete entity recognition. Finally, the pharmacogenomics knowledge model was described in detail.

**Results:**

In entity recognition tasks, the Bidirectional Encoder Representations from Transformers–conditional random field model achieved better performance with micro-F1 score of 85.12%. The pharmacogenomics knowledge model in our study included 5 semantic types: drug, gene, disease, precise medication (population, daily dose, dose form, frequency, etc), and adverse reaction. Meanwhile, 26 semantic relationships were defined in detail. Taking melanoma caused by a *BRAF* gene mutation into consideration, the pharmacogenomics knowledge model covered 7 related drugs and 4846 triples were established in this case. All the corpora, relationship definitions, and triples were made publically available.

**Conclusions:**

We highlighted the pharmacogenomics knowledge model as a scalable framework for clinicians and clinical pharmacists to adjust drug dosage according to patient-specific genetic variation, and for pharmaceutical researchers to develop new drugs. In the future, a series of other antitumor drugs and automatic relation extractions will be taken into consideration to further enhance our framework with more PGx linked data.

## Introduction

### Pharmacogenomics

The field of pharmacogenomics (PGx) has developed rapidly since the initial scientific discoveries of genetic characteristics affecting individual response to drugs or other agents [1]. Through these years of development, PGx aims at understanding how genetic variants influence drug efficacy and toxicity. It combines pharmacology (the science of drugs) and genomics (the study of genes and their functions), and is certain to improve new drug development and precision medication. Such studies can reveal how genetic variation across individuals affects a drug’s pharmacokinetics and pharmacodynamics [2]. Many drugs do not work the same way for everyone. Consequently, PGx is often considered one of the most actionable areas of the personalized medicine paradigm [3].

As of June 2019, more than 190 drugs [4] approved by the US Food and Drug Administration (FDA) clearly stated on in their medical specifications that they need to be deployed with greater precision based on individual genotype. The introduction of targeted drugs and targeted therapies provides a more feasible and effective way for cancer treatment, improves drug efficacy, and reduces adverse reactions. Therefore, studies of new therapies related to PGx such as drug combinations and new drug discoveries [5] have become increasingly popular. A typical case of repurposing drugs is afatinib (40 mg q.d.), which was introduced [6] for treating lung cancer after *NGR1* gene fusion.

### Named Entity Recognition

Named entity recognition (NER) is a basic tool for natural language processing (NLP) tasks such as information extraction, question answering system, syntactic analysis, and machine translation. Its main goal is identifying entities with specific meaning in the text, mainly including people’s names, place names, organization names, proper nouns, etc. It is the foundation of identifying semantic relationships between entities and filling a knowledge base.

The common statistical models of NER mainly include the Hidden Markov Model [7] and the conditional random field (CRF) [8]. In recent years, neural network deep learning methods based on the development of word vector technology, such as the convolutional neural network (CNN) [9] and the recurrent neural network (RNN), have made a great breakthrough in the field of NLP. After that, long short-term memory (LSTM) [10] added a memory cell to RNN, to overcome the problem of gradient explosion and gradient disappearance. Bidirectional RNN [11] adopts a double-layer RNN structure, which can collect forward and backward information at the same time.

In 2018, Devlin et al [12] from Google AI Language proposed the Bidirectional Encoder Representations from Transformers (BERT) which provided outstanding performance in 11 NLP tasks, opening a new era for NLP. Similar to the general pretraining 2-stage training method, BERT uses the language model for pretraining as the first stage. In the second stage, it fine-tunes for downstream tasks, and achieves the best results in multiple NLP tasks. The BERT–CRF model [13] and multilingual BERT model [14] were trained on different languages such as Portuguese and the F1 score was ultimately improved. Today, the BERT model has also been applied in biomedical research. BERT-based models were investigated for their effectiveness in biomedical and clinical entity normalization, and achieved state-of-the-art performance on large-scale electronic health record notes [15] and online corpus [16]. The BioBERT model [17] for biomedical text mining tasks and the ClinicalBERT [18] for clinical notes were also introduced and outperformed previous models.

### Biomedical Knowledge Representation

The Knowledge Representation Model can be understood as a structured set of directed graphs, in which the nodes of the graph represent entities or concepts, while the edges represent the semantic relationship between entities or concepts. During the development of the knowledge representation, sematic networks, ontology, and knowledge graphs/models are most commonly used in the field of biomedical science.

A semantic network [19], or frame network, is a knowledge base that represents semantic relations between concepts in a network.

An ontology is a formal explicit description of concepts in a domain, properties of each concept, various features and attributes, and restrictions on these properties [20]. The Drug Target Ontology [21] provided a framework and formal classification, which included related information between protein, gene, protein domain, binding site, small-molecule drug, mechanism of action, and many other types of information. Dumontier and Villanuevarosales [22] constructed a lightweight ontology, Pharmacogenomics Ontology, based on Pharmacogenomics Knowledge Base (PharmGKB) data, which contains 40 core concepts, involving phenotype, genotype, and drug therapy.

A knowledge graph/model emphasizes data cleaning and knowledge fusion, and its essence is a semantic network, which allows access to knowledge inference. Since this concept was put forward by Google in 2012 [23], researchers have conducted a series of discussions and research aimed at intelligent retrieval. High-quality heterogeneous graphs such as the Safe Medicine Recommendation (SMR) [24] and KnowLife [25] contain entities and relationships between disease, medicine, patient, gene, organ, and other biomedical entities constructed by bridging electronic medical records, ICD-9, DrugBank, electronic health record [26], and other databases, which leads to more hidden relationships.

Above all, the knowledge graph/model technology provides a means to extract structured knowledge from massive texts and images. It has broad applications in biomedical field and can promote intelligent semantic retrieval, medical questions and answers, clinical decision support, and many other scenarios.

### Related Works

With the rapid growth and accumulation of massive PGx data, there is an increasing need for scientific data collecting, organizing, modeling, and mining. These data reflect a hierarchy of relationships and detailed information between biomedical entities. Currently, the semantic types and relationships involved in PGx knowledge representation are usually limited to drug, gene, and disease.

#### Drug–Gene Target Treatment

Drug2Gene [27] was a knowledge base combining information on compound, drug, gene, and protein from 19 publicly available databases. Sun et al [28] designed a computational workflow to construct drug-target networks including drugs, genes, and diseases from different knowledge bases.

#### Drug–Gene–Drug Interaction

Bo et al [29] extracted drug–gene–drug interactions from biomedical literature using the bidirectional LSTM (Bi-LSTM) model by combining biomedical resources with lexical information and entity position information. Coulet et al [30] instantiated a description logics knowledge base to identify gene variant–drug response associations.

#### Drug–Gene–Phenotype Relationship

Dalleau et al [31] assembled a set of linked PGx data from 6 distinct resources such as DisGeNET [32] and ClinVar [33].

#### Disease–Chemical–Gene Relationship

Kim et al developed DigSee [34] for disease–gene relationships and DigChem [35] for disease–gene–chemical relationships from biomedical literature abstracts at a PubMed scale.

However, there currently exist no in-depth explorations and descriptions of personalized medication, such as drug usage, dosage adjustment, and applicable population. Therefore, there is significance in applying the knowledge model to the field of PGx in further study, which will assist clinicians and clinical pharmacists in precise medication.

### Objective

In this study, we proposed the following 2 objects:

1. We aimed to present a pharmacogenomics knowledge model consisting of 5 semantic types related to PGx and precision medication, and also give definitions of relationships between these entities. The model mostly focuses on anticancer drugs, drug usage, and adjustments of daily dosage.
2. We aimed to semiautomatically construct PGx corpora, which are relatively rare in the existing research, and make them open access. The NLP algorithms for PGx NER were also trained for facilitating corpus annotation.

## Methods

### Study Steps

**Figure 1:**
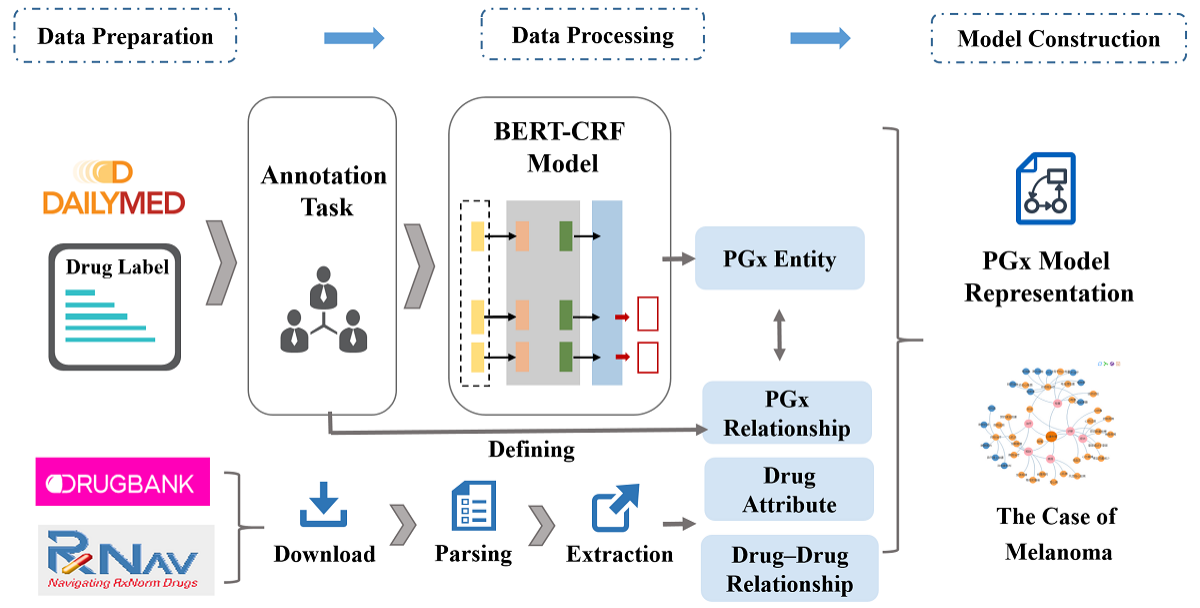
The framework of our study.

There are 3 main steps in our study (Figure 1).

1. Data preparation: Data related to PGx were collected from DailyMed, DrugBank, and RxNorm.
2. Data processing: Manual annotation for PGx entities and relationships were applied to drug labels in PDF/XML format from DailyMed. The BERT–CRF model were trained for entity recognition in this study. Data from DrugBank and RxNorm were also downloaded, parsed, and extracted for more drug attributes and relationships.
3. Model construction: The PGx knowledge model was described in this aspect based on the entities and relationships extraction. Melanoma was also used as an example to verify the accuracy and validity of our model.

### Data Preparation

Data related to PGx need to be collected and integrated in this study, which are currently stored in DrugBank, PharmGKB, Comparative Toxicogenomics Database (CTD), RxNorm, and other databases. Based on the pharmacogenomics knowledge model built in our study, we chose the following 3 data sources to accomplish data crawling and data preparation.

#### DailyMed

The text of drug labels was obtained from DailyMed, which is a free drug information resource [36] provided by the US National Library of Medicine (NLM). It consists of digitized versions of drug labels as submitted to the US FDA. DailyMed was of special interest because of its comprehensive coverage, open availability, and the package inserts’ combination of format consistency and rich detail. Drug labels in DailyMed give a detailed description of drugs’ indications and usage, adverse reaction, and applicable population, especially the dosage, dose form, and dosage adjustment. We downloaded 4067 drug labels randomly for pretraining tasks and 190 drug labels in the table of PGx biomarkers for annotation tasks.

#### DrugBank

DrugBank is a unique bioinformatics and cheminformatics resource that combines detailed drug (ie, chemical, pharmacological, and pharmaceutical) data with comprehensive drug target (ie, sequence, structure, and pathway) information [37] provided by the University of Alberta. The latest release of DrugBank (version 5.1.4, released July 2, 2019) was parsed in this paper for drug attributes such as drug name, description, chemical formula, molecular weight, drug approval status, and so on.

#### RxNorm

RxNorm [38] provides a suite of standards for clinical drugs in the form of “Ingredient–Strength–Dose Form–Brand name,” and is designed by NLM for the electronic exchange of clinical health information. Several attributes and drug–drug interactions of precise medication were selected from RxNorm, such as daily dose, dose form, and frequency as attributes, and has_dose_form, dose_form_of as relationships.

### Annotation Task

We recruited 3 annotators, all of whom had a medical training background and curation experience. Each drug label was annotated independently by 2 annotators (ie, double annotation). Differences were resolved by a third and senior annotator. Besides this, we measured agreement of relationship annotations using the *F* score to assess consistency.

Because all 190 drug labels in the FDA table of PGx biomarkers [4] are in PDF format, the annotator needed to convert all of them into an editable format such as .txt (Notepad or other word processors) or .doc/.docx (Microsoft Word) before annotation.

**Figure 2:**
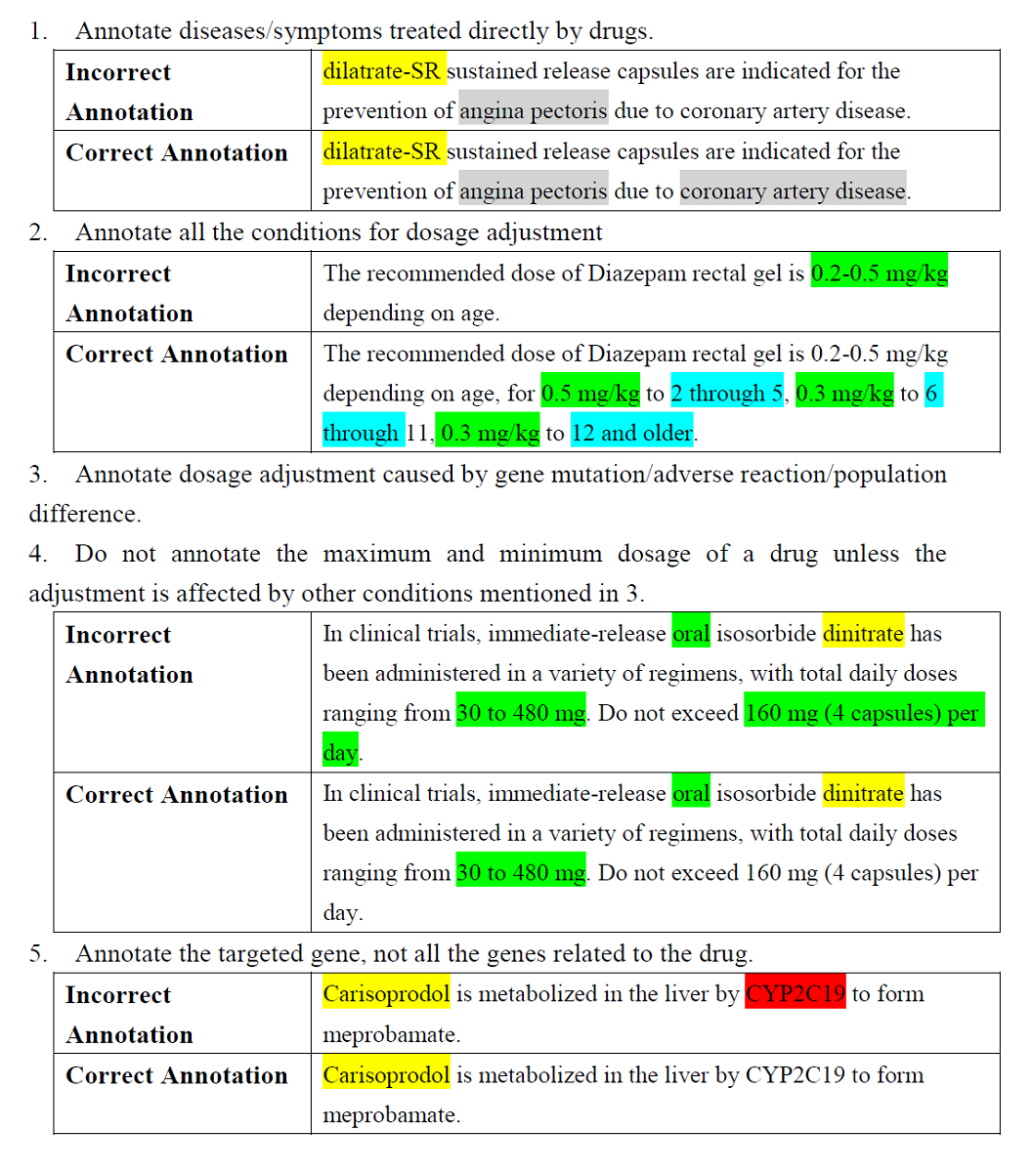
Annotation guidelines.

The main tasks involved in the annotation stage were the recognition of semantic types and semantic relationships from drug labels sections, including “Indications and Usage,” “Dosage and Administration,” “Use in Specific Populations,” “Warnings and Precautions,” and “Adverse Reactions.” For semantic types, different highlighted colors represented different entities according to the frame of the PGx knowledge model. In this work, drug was annotated in yellow, gene was annotated in red, disease was annotated in gray, dosage and dose form were annotated in green, adverse reaction was annotated in purple, and population was annotated in blue. For semantic relationships, the more important and difficult section, annotators read the drug labels and recorded the relation descriptions between diseases and drugs, diseases and genes, diseases and diseases, drugs and genes, drugs and drugs, and drugs and dosage manually. This formed the basis of relationship definition in the follow-up work. Before annotation, we also indicated the annotation guidelines, see in Figure 2.

**Figure 3:**
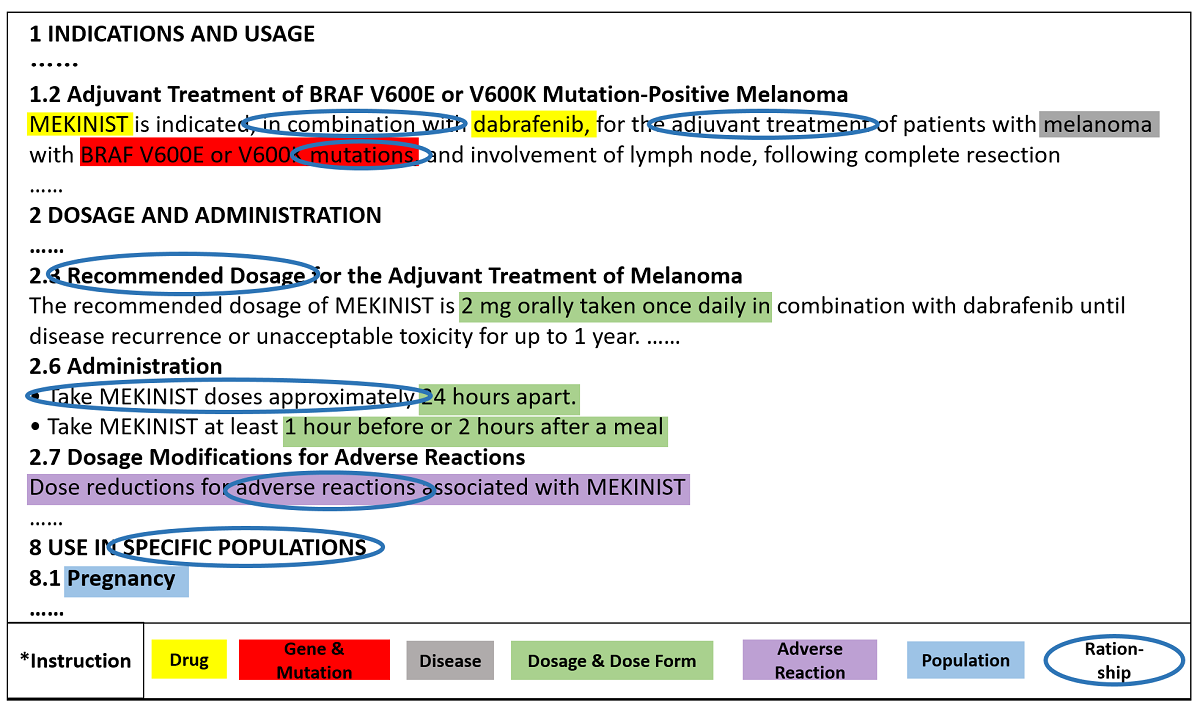
Annotation example of MEKINIST.

An example of drug label annotation is shown in Figure 3. Finally, all the annotated semantic types and relationships were recorded in a structured database designed in advance.

### BERT–CRF for NER

After the annotation of entities, we applied the BERT–CRF model for NER. The CRF model and BERT–Bi-LSTM–CRF model were also trained in our study as a comparison.

**Figure 4:**
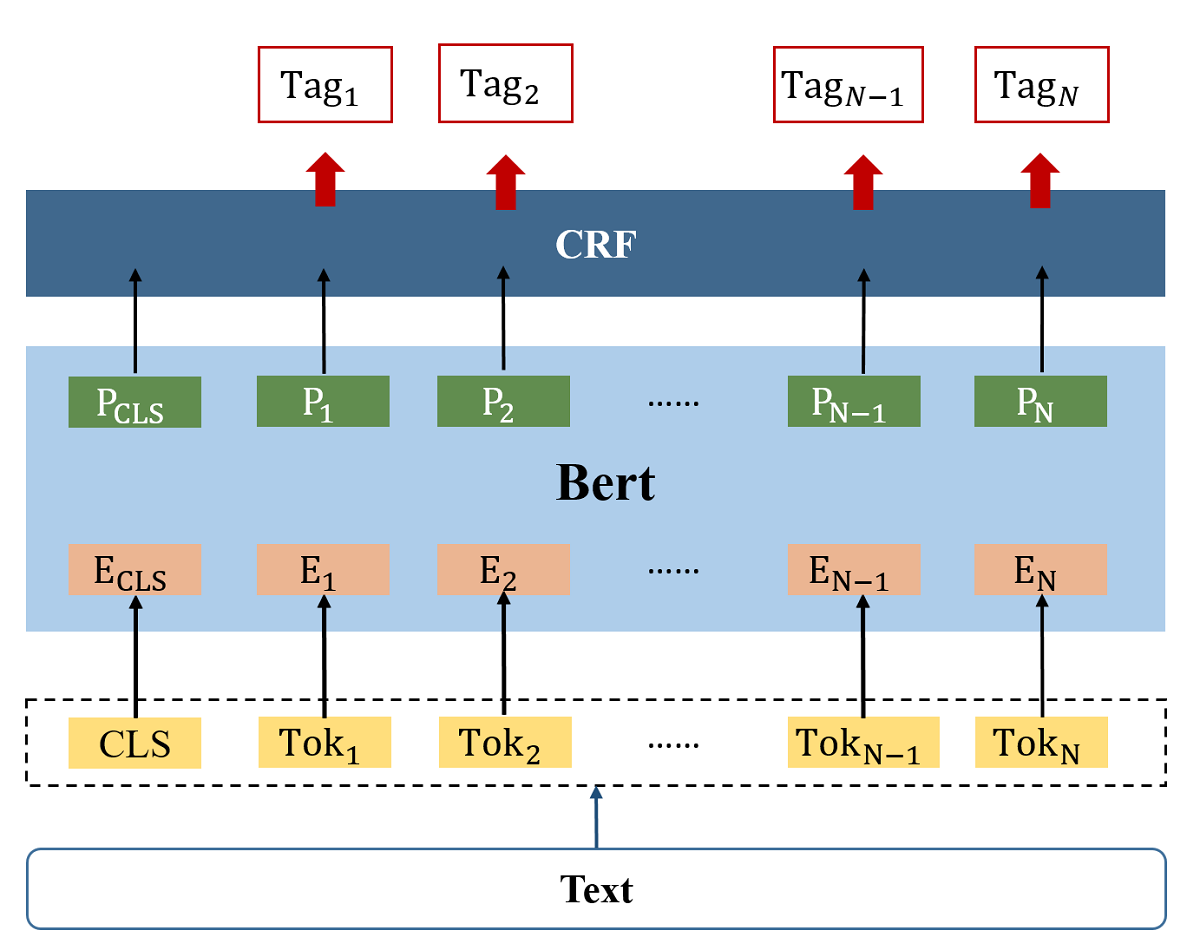
BERT–CRF architecture. BERT: Bidirectional Encoder Representations from Transformers; CRF: Conditional Random Field.

The BERT–CRF architecture was composed of 4 sections: the input layer, the pretraining model, the full connection layer, and the CRF layer, which assigns a tag to each word based on its context in the output (Figure 4). We feed a sentence to the architecture to obtain contextual BERT embedding for each word as {Tok_1_,...,Tok_N_} The context could be captured via many attention heads in each of its layers as well. These embeddings were then transported to a CRF layer to obtain the tag as {Tag_1_,...,Tag_N_} for each word block.

The BERT-Base Multilingual, which has 110M parameters, was used in this NER task. We set the training batch size to 32, the max_seq to 80, and the learning rate to 0.00001. A total of 10 epochs were trained in each iteration to ensure model convergence. Other parameters related to BERT are set to default values. The dropout rate was set to 0.9 in fully connected layers to prevent over fitting. The transfer matrix in CRF is also left for the model to learn. The transfer matrix in the CRF layer was learned by the model itself. Importantly, the Bi-LSTM layer was added in this architecture before feeding the tweet-level representation into the CRF layer, to compare the performance between BERT–CRF with Bi-LSTM and without Bi-LSTM.

### Model Representation

We extended the semantic types of our model from 3 common types of drug, gene, and disease to 5 types: drug, gene (gene name, gene mutation), disease (disease name, position, etc), precise medication (population, daily dose, dose form, frequency, take time for, take with a meal or not, etc), and adverse reaction.

All the semantic types and attributes covered in pharmacogenomics knowledge model are shown in Table 1.

The entities model in pharmacogenomics knowledge model was defined and EID represented the unique identifier for entities

     Entity={EID*,TERM*,Source,SEMANTICType*} (1)

The relationships model in pharmacogenomics knowledge model was defined and RID represented the unique identifier for relationships

     Relation={RID*,Relationship*,Domain*,

          Range*,Definition,TreeNumber*} (2)

The whole pharmacogenomics knowledge model can be represented as the risk factors of precision medication for cancers. In this model, disease (C, especially for cancer in this paper) is usually caused by gene mutations (G), which decided the target drug (Dr) for treatment.

     Dr = F(C,G) (3)

During treatment, routine dosage/dose form (Ds) has been already offered by the FDA drug labels. However, it differs when the patient has an adverse reaction (A) or the disease occurs in special groups (P) such as pregnancy, lactation, pediatric, geriatric. Assuming that the 4 factors are independent in some cases, each factor can effect dosage/dose form separately.

     Ds = F(Dr,G,A,P) (4)

Above all, gene mutation, disease, adverse reaction, and patient populations are the risk factors in pharmacogenomics knowledge model of drugs to be used, and suitable dosage and dose form especially.

     Dr, Ds=F(C,G,A,P) (5)

**Table 1:** Semantic types and attributes in the knowledge model.

Semantic Type	Entity/Attribute
Drug	Drug Name, Description, Chemical Formula, Molecular Weight, Drug Approval Status, CAS^a^, UNII^b^, Pharmacology Indication
Gene	Gene name, Mutation
Disease	Disease Name, Position
Adverse Reaction	N/A^c^
Population	Pediatric Use Population, Applicable Population, Gender, Age, Race
Drug Use	Daily dose, Dose form, Frequency, Take time for, Take with a meal or not, etc

^a^CAS: Chemical Abstracts Service Number.

^b^UNII: Unique Ingredient Identifier.

^c^N/A: not available.

## Results

### Data Set Overview

In this paper, we have collected 4067 drug labels in XML format downloaded from DailyMed as pretraining data for the BERT–CRF architecture, and 190 drug labels after annotation for model representation in which 90% (n=171) form the training set and 10% (n=19) form the test set, randomly assigned. Statistics-annotated corpus are presented in Table 2. Besides, the number of unique unigrams were 2216 in the training set and 829 in the test set; the number of unique bigrams were 120,705 in the training set and 18,851 in the test set.

**Table 2:** Number of entities in training and test sets.

Entity	Number of entities in the training set	Number of entities in the test set
Drug	76	31
Gene	60	26
Disease	94	33
Body_Part	23	7
Daily_Dose	99	27
Dose_Form	16	8
Frequency	32	12
Adverse_Reaction	372	77

### Performance of Named Entity Recognition

Three basic models are compared, with the specific results shown in Table 3 in which minor averaging for the F1 score was used. The BERT–CRF model achieved better performance than the other 2 models in this task. In some recent studies, the full connectivity layer was done by the Bi-LSTM layer, which ultimately resulted in the BERT–Bi-LSTM–CRF model. However, the BERT–Bi-LSTM–CRF model presented a more complex structure and slower training speed than BERT–CRF. Besides this, there was a little difference of 2% between these 2 models, so BERT–CRF was selected in our study. The BERT–CRF model showed a high F1 score in drug, dose form, and body part, but a low F1 score in daily dose and disease, shown in Table 4. However, these performances were only for the PGx corpus built semiautomatically in this work, and the 3 basic models may present different results in other studies with large-scale corpora.

**Table 3:** Performance of the models.

Model	Precision (%)	Recall (%)	F1 (%)
CRF^a^	88.03	73.57	80.16
BERT–CRF^b^	85.12	85.12	85.12
BERT–Bi-LSTM–CRF^c^	85.22	81.00	83.05

^a^CRF: Conditional Random Field.

^b^BERT: Bidirectional Encoder Representations from Transformers

^c^Bi-LSTM: Bidirectional Long Short-Term Memory.

**Table 4:** Performance of the semantic type.

Semantic type	F1		
	CRF^a^ (%)	BERT–Bi-LSTM–CRF^b,c^ (%)	BERT–CRF (%)
Drug	94.12	94.12	100.00
Gene	66.67	80.00	71.43
Disease	61.54	66.67	57.14
Body_Part	57.14	57.15	85.71
Daily_Dose	31.58	31.58	42.11
Dose_Form	100.00	100.00	100.00
Frequency	62.50	75.00	75.00
Adverse Reaction	68.15	79.00	73.74

^a^CRF: Conditional Random Field.

^b^BERT: Bidirectional Encoder Representations from Transformers

^c^Bi-LSTM: Bidirectional Long Short-Term Memory.

### Semantic Relationships Extraction

Because this study required a high accuracy of relationship extraction, we adopted a manual method in this task. Descriptions of semantic relationships were normalized at the same time during annotation, such as “in combination with” = “synergized by,” “recommended dosage” = “routine dosage.” The normalized descriptions are presented in Table 5. The other expressions in drug labels were stored as synonyms in our study at the same time. In order to make the pharmacogenomics knowledge model be more portable, several semantic relationships were extended, such as “is biomarker-efficacy of,” “is biomarker-prognosis of.”

In the end, 26 kinds of semantic relationships were extracted, and the consistency of the entity relationship annotation was 78.55%. Among them, there were 14 first-level semantic relationships and 12 second-level semantic relationships. Each kind of semantic relationships has been defined in detail, as shown in the accessory document.

**Table 5:** Examples of semantic relationship–normalized description.

Normalized description	Expressions in drug labels
Treats	for the prevention of, for relief of the signs and symptoms, for the treatment of, for the prevention of, as monotherapy of
Synergized by	in combination with, coadministered with
Antagonized by	avoid concurrent administration of, avoid concomitant use of
Have dosage	total daily doses, recommended dosage
Have mutation	with *** mutation, the presence of *** mutation, be homozygous for

### Pharmacogenomics Knowledge Model

**Figure 5:**
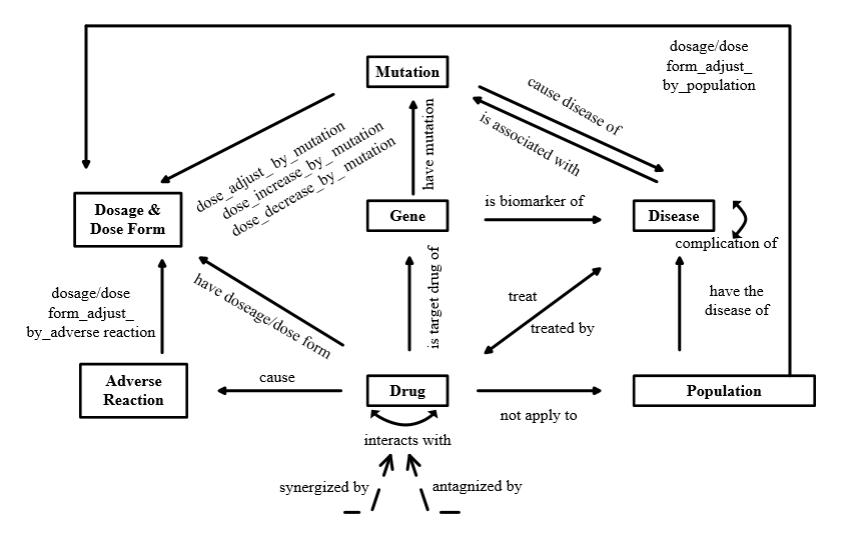
Overview of pharmacogenomics knowledge model.

Based on the entity recognition and relationship definitions mentioned above, the pharmacogenomics knowledge model is presented as Figure 5.

### The Case of Melanoma

Melanoma is a malignant neoplasm derived from cells that are capable of forming melanin, which may occur in the skin of any part of body. It frequently metastasizes widely, and the regional lymph nodes, liver, lungs, and brain are likely to be involved. The incidence of malignant skin melanomas is rising rapidly in all parts of the world. Therefore, melanoma, which is caused by *BRAF* gene mutation, was taken as an example to verify our model.

Seven drugs were included in the cases: binimetinib, cobimetinib, dabrafenib, encorafenib, nivolumab, trametinib, and vemurafenib. Most were newly indicated for the treatment of unresectable or metastatic melanoma with *BRAF* V600E or V600K mutations, as detected by FDA-approved tests in 2018. Among them, dabrafenib, encorafenib, and vemurafenib are targeted drugs for *BRAF* gene mutations.

**Figure 6:**
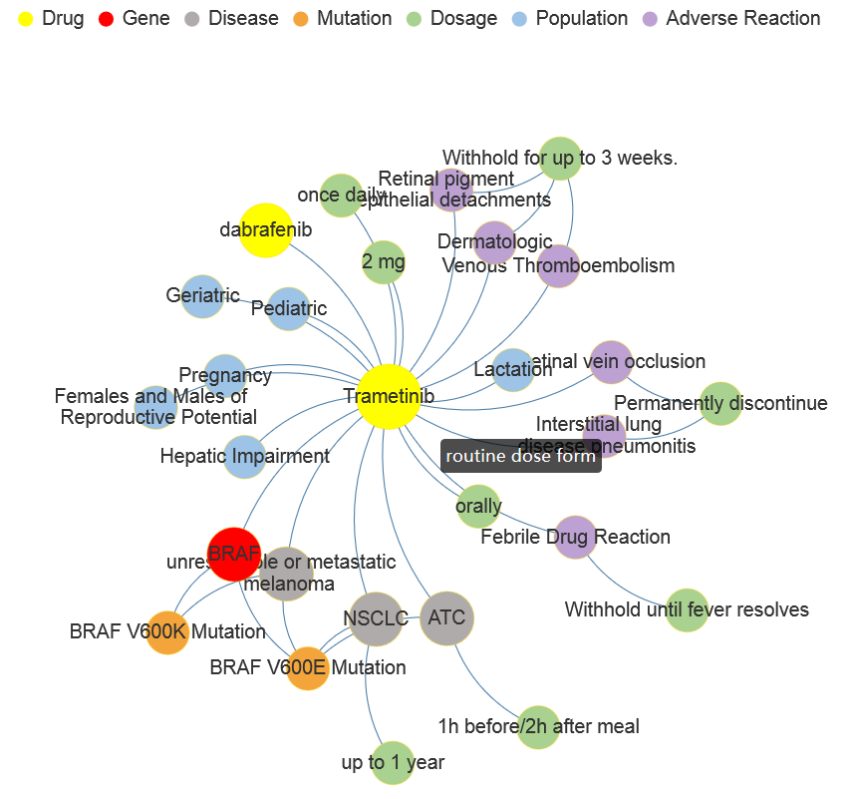
An example of pharmacogenomics knowledge model data visualization.

By researching the 7 drugs, 4846 triples were established in the pharmacogenomics knowledge model of melanoma, among them 4713 triples were drug–drug relationships, 41 were drug–adverse reaction, 30 were drug–dosage, 24 were adverse reaction–dosage, 22 were drug–disease, 7 were drug–gene, 4 were drug–population, 2 were gene–mutation, and 3 were gene–disease. An example of data visualization of trametinib can be seen in Figure 6. Relationships can be displayed when the mouse hovers over the joint(s).

### Data Set Access

**Figure 7:**
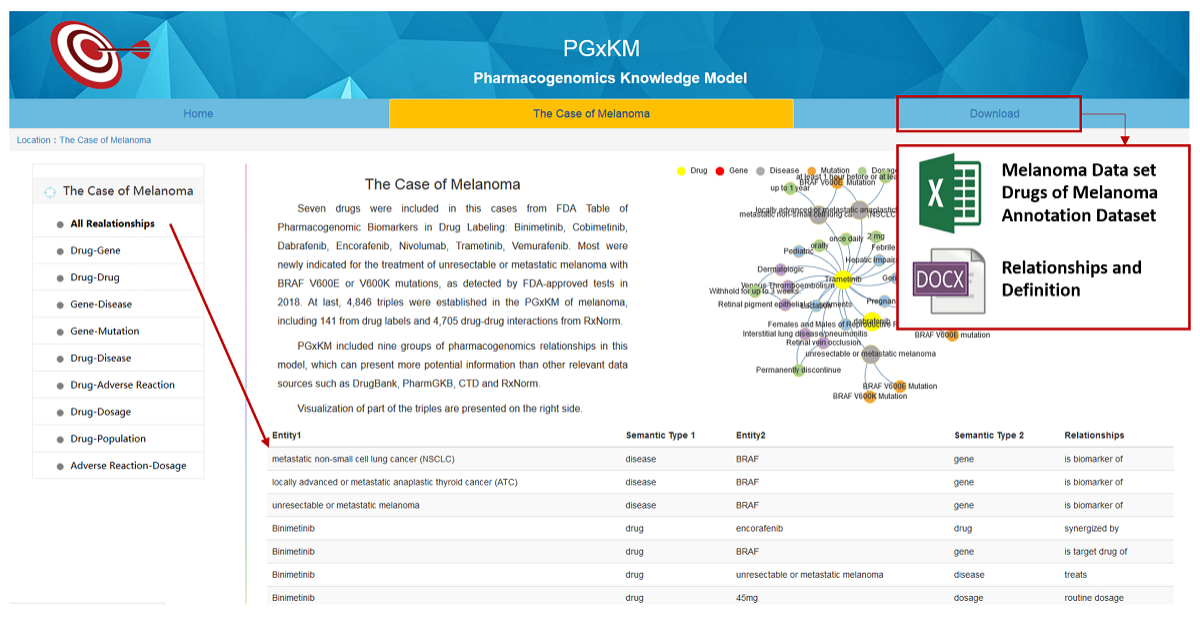
User interface of pharmacogenomics knowledge model data set.

We provided a user-friendly interface [39] that enables users to access the pharmacogenomics knowledge model data set (Figure 7). In the “Home” page, users can learn basic information and purpose of this knowledge model. On “The Case of Melanoma” page, users can obtain all the triples in melanoma cases and browse the triples by different groups of relationships. Visualization of the triples are presented as well. On the “Download” page, users can download the melanoma data set, drug attribute data set, and annotated data set in Microsoft Excel format, as well as the relationships and definition document in Microsoft Word format for the user’s convenience.

## Discussion

### Potential Relationships in Pharmacogenomics Knowledge Model

The pharmacogenomics knowledge model constructed in this paper reveals hidden relationships between drug, gene, disease, precise medication, and adverse reaction. Trametinib is used as an example, which is a kinase inhibitor indicated as a single agent for the treatment of BRAF-inhibitor treatment-naïve patients with unresectable or metastatic melanoma with *BRAF* V600E or V600K mutations as detected by an FDA-approved test. The recommended dosage is 2 mg orally once daily, and should be taken at least 1 hour before or at least 2 hours after a meal. However, we recognized from pharmacogenomics knowledge model that more careful attention should be paid to dosing schedules, when medication experience changes or other side effects occur. That is to say, trametinib needs to be stopped permanently in case of fever or interstitial lung disease, taken 1-2 hours before meals in case of metastatic thyroid cancer, and once a day in case of liver injury.

### Comparison With Relevant Data Sources

The pharmacogenomics knowledge model included 9 groups of PGx relationships in this model, which can present more potential information than other relevant data sources such as DrugBank, PharmGKB, CTD, and RxNorm, as shown in Table 6.

**Table 6:** Comparison between pharmacogenomics data sources.

Relationships	DrugBank	PharmGKB^d^	CTD^e^	RxNorm^f^	PGxKM^g^
Drug–Gene	√^a^	√	√	—	√
Drug–Drug	√	√*^b^	—	—	√
Gene–Disease	—^c^		√	—	√
Gene–Mutation	—	√	—	—	√
Drug–Disease	√	√*	√	—	√
Drug–Adverse Reaction	—	—	—	—	√
Drug–Dosage	√	—	—	√	√
Drug–Population	—	—	—	—	√
Adverse Reaction–Dosage	—	—	—	—	√

^a^Have structured data and can be downloaded in the web set.

^b^Have information (unstructured data) for such relationships in the web set.

^c^Have no information for such relationships in the web set.

^d^PharmGKB: Pharmacogenomics Knowledge Base.

^e^CTD: Comparative Toxicogenomics Database.

^f^RxNorm: drug data interaction standard in American Clinical Information System

^g^PGxKM: pharmacogenomics knowledge model.

### Limitations and Future Studies

However, there are still some limitations in our study. First, this study aimed to build a pharmacogenomics knowledge model and semiautomatically annotate the corpus using the existing NLP tools. However, we did not validate the feasibility of NLP tools or compare the NLP performance using a benchmark data set, such as clinical records from the Third i2b2 Workshop on NLP Challenges [40] or LabeledIn [41], of labeled indications for human drugs. Our future research will explore BERT–CRF model verification on other standard drug corporas. Second, relation extraction was manually done by the 3 annotators which will place restrictions on the application of pharmacogenomics knowledge model, and an evaluation of automatic relation extraction will be conducted in the future. Common relation extraction methods such as CNN, LSTM, and BERT method will be used to improve extraction efficiency.

In future studies, we also plan to do the following jobs to improve our research. First, a series of other antitumor drugs will be taken into consideration to fill up our framework, such as ceritinib and afatinib for non–small-cell lung cancer. Second, linked data can also be extended to other sources, such as CTD, PharmGKB, and DisGeNET. We hope that this knowledge model for PGx interactions could serve as a framework and a resource for future drug research and development.

### Conclusions

A pharmacogenomics knowledge model was constructed for precision medication in our research, which reflected the multidimensional relationships between drug, gene, disease, as well as relationships from gene to drug to dosage or frequency associations. Extraction task for PGx entities has been done using the BERT–CRF model with F1 score of 85.12%. Our pharmacogenomics knowledge model contained 5 semantic types (drug, gene, disease, precise medication, and adverse reaction) and 26 semantic relationships had been defined in detail. Using melanoma caused by *BRAF* gene mutation as an example, we verified the feasibility of this model using the FDA’s drug labels and relevant linked data. Finally, we highlighted this knowledge model as a scalable framework for clinicians and clinical pharmacists to adjust drug dosage according to patient-specific genetic variation, and to support pharmaceutical researchers during new drug discoveries.

## Abbreviations

ATC: Anaplastic thyroid cancer
BERT: Bidirectional Encoder Representations from Transformers
Bi-LSTM: bidirectional long short-term memory
CRF: conditional random field
CTD: the Comparative Toxicogenomics Database
FDA: the US Food and Drug Administration
NLM: the US National Library of Medicine
PGx: pharmacogenomics
PharmGKB: Pharmacogenomics Knowledge Base
